# Caregivers’ perception about their participation in a community-based exercise intervention - Body & Brain Project

**DOI:** 10.1093/geroni/igab046.3245

**Published:** 2021-12-17

**Authors:** Oscar Ribeiro, Pedro Marques, Duarte Barros, Paula Silva, Joana Carvalho, Flávia Borges-Machado

**Affiliations:** 1 University of Aveiro, University of Aveiro, Aveiro, Portugal; 2 Department of Education and Psychology, University of Aveiro, University of Aveiro, Aveiro, Portugal; 3 Research Centre in Physical Activity, Health and Leisure (CIAFEL), Faculty of Sports, University of Porto, University of Porto, Porto, Portugal

## Abstract

Evidence is scarce on caregivers’ perception regarding their participation in exercise interventions targeting individuals with neurocognitive disorder (NCD). This study aims to investigate the views of family caregivers of people with NCD about taking part in a community-based physical exercise intervention with their care-recipients. Twenty caregivers (N Male: 13; 66.5 ± 14.39 years old; age range: 36-88) answered to a semi-structured interview conducted by telephone about their perception on participating or not as class members of a 6-month multicomponent training intervention. Transcribed data from the interview were analyzed through thematic analysis. Main themes regard their perceived key-role in the care recipients’ participation, which included knowing their limitations, making them feel accompanied and motivated, and the possibility of providing comfort and tranquility throughout the intervention. Caregivers also mentioned the possibility of fulfilling own needs for physical activity and being engaged in new experiences. Disturbing the care recipients’ involvement and performance, the opportunity for respite during the sessions’ time, and being enrolled in the program only in specific moments or by telephone were also mentioned. Findings highlight the inclusive perspective of caregivers to take part of exercise programs designed for people with NCD, not only due to their decisive role on care-recipients engagement but also due to the associated (in-/)direct personal benefits. This data may be useful for planning and prescribing future community-based exercise interventions for NCD caregiving dyads. Trial registration: ClinicalTrials.gov - NCT04095962. Supported by FCT: “Body and Brain” (POCI-01-0145-FEDER-031808), CIAFEL (FCT/UIDB/00617/2020), and Ph.D. Grants (SFRH/BD/136635/2018) to FM (2020.05911.BD) to DB.

